# PilotCareTrans Net: an EEG data-driven transformer for pilot health monitoring

**DOI:** 10.3389/fnhum.2025.1503228

**Published:** 2025-01-29

**Authors:** Kun Zhao, Xueying Guo

**Affiliations:** ^1^Physical Education Department, Civil Aviation University of China, Tianjin, China; ^2^Computer Science and Technology College, Civil Aviation University of China, Tianjin, China

**Keywords:** pilot health monitoring, transformer-based model, EEG data analysis, temporal dynamics, cognitive health intervention

## Abstract

**Introduction:**

In high-stakes environments such as aviation, monitoring cognitive, and mental health is crucial, with electroencephalogram (EEG) data emerging as a keytool for this purpose. However traditional methods like linear models Long Short-Term Memory (LSTM), and Gated Recurrent Unit (GRU) architectures often struggle to capture the complex, non-linear temporal dependencies in EEG signals. These approaches typically fail to integrate multi-scale features effectively, resulting in suboptimal health intervention decisions, especially in dynamic, high-pressure environments like pilot training.

**Methods:**

To overcome these challenges, this study introduces PilotCareTrans Net, a novel Transformer-based model designed for health intervention decision-making in aviation students. The model incorporates dynamic attention mechanisms, temporal convolutional layers, and multi-scale feature integration, enabling it to capture intricate temporal dynamics in EEG data more effectively. PilotCareTrans Net was evaluated on multiple public EEG datasets, including MODA, STEW, SJTUEmotion EEG, and Sleep-EDF, where it outperformed state-of-the-art models in key metrics.

**Results and discussion:**

The experimental results demonstrate the model's ability to not only enhance prediction accuracy but also reduce computational complexity, making it suitable for real-time applications in resource-constrained settings. These findings indicate that PilotCareTrans Net holds significant potential for improving cognitive health monitoring and intervention strategies in aviation, thereby contributing to enhanced safety and performance in critical environments.

## 1 Introduction

Temporal prediction in health intervention decision-making for aviation students has become a crucial area of research, driven by the increasing emphasis on monitoring cognitive and psychological health in aviation training. Aviation students face intense mental pressure and complex operational demands during training, where changes in their health status not only impact training effectiveness but can also pose significant safety risks (Zhang J. et al., 2024). Therefore, accurately predicting the health status of aviation students and implementing timely interventions is essential. This research not only enhances the overall health levels of aviation students but also provides robust data support for airlines and flight schools, ultimately contributing to improved flight safety (Kulkarni et al., 2024).

To address the limitations of traditional methods in predicting health status, early research primarily relied on statistical approaches, which were seen as robust tools for forecasting based on historical data. These techniques aimed to predict future health conditions by analyzing trends, patterns, and cyclical variations in past records. Among the commonly employed methods were time series analysis models such as AutoRegressive Integrated Moving Average (ARIMA), which focused on the temporal dependencies within data to predict future states based on observed sequences (Anand, 2021). Additionally, regression analysis was frequently utilized to explore the relationships between variables, allowing researchers to identify potential predictors of health outcomes. However, while these methods provided reliable predictions for relatively simple, linear, and regularly structured time series data, they exhibited significant limitations when confronted with more complex, nonlinear relationships inherent in many health-related datasets. In particular, these techniques often struggled to capture the intricate dependencies between variables in multivariate contexts, where interactions between different factors could significantly influence the outcome. Furthermore, these statistical methods proved inadequate in handling noisy or incomplete data, which is often the case in real-world applications. This issue becomes particularly pronounced in the context of dynamic environments such as aviation training, where irregularities in data collection, inconsistencies, and outliers are frequent. The need for data completeness and regularity in traditional statistical approaches rendered them less effective for applications where real-time health monitoring is essential, and the data is inherently unpredictable and subject to abrupt changes (Zhao et al., 2024). As a result, more advanced techniques, such as machine learning models, have emerged as more effective alternatives to address these challenges in predicting health status in such complex environments.

To overcome the shortcomings of traditional statistical methods, machine learning methods and early neural network techniques were introduced for health status temporal prediction tasks. Compared to traditional statistical approaches, machine learning methods such as Support Vector Machines (SVM) (Kurniawan et al., 2020), Decision Trees, and Random Forests provided more flexible model structures, capable of capturing more complex data patterns, thereby improving prediction accuracy to some extent (Anand, 2021). For example, SVM finds the optimal hyperplane in high-dimensional space to separate different classes, making it suitable for handling complex classification tasks. Decision Trees, on the other hand, create predictive models through hierarchical decision rules, while Random Forests enhance model robustness by building multiple tree models and aggregating their results. While these methods effectively improved predictive performance, they still had limitations, particularly when dealing with temporal dependencies and complex multivariate data. Furthermore, early neural network models, such as Multilayer Perceptrons (MLP) (Zhang J. et al., 2024) and basic Recurrent Neural Networks (RNN) (Ouchene and Bessou, 2022), were also applied to temporal prediction tasks. MLP, a type of feedforward neural network, consists of multilayer connections of neurons and can make predictions by learning nonlinear relationships within data through repeated iterations. However, because MLP lacks a memory mechanism to handle temporal data, it is not ideal for tasks where past information needs to influence future predictions. To address this issue, Recurrent Neural Networks (RNN) were introduced. RNNs incorporate loops in their network architecture, enabling the model to retain a hidden state that evolves over time. This mechanism allows RNNs to learn dependencies between sequential data points, making them more suitable for temporal prediction tasks (Ouchene and Bessou, 2022). However, RNNs also have some shortcomings, particularly when dealing with long-term dependencies. The vanishing gradient problem makes it difficult for the model to learn and retain useful information over longer sequences. Additionally, handling high-dimensional data significantly increases model complexity and computational demands, which limits its practical applications to some extent. Despite these drawbacks, these machine learning methods and early neural network techniques laid the groundwork for the development of more sophisticated deep learning models.

To address the limitations of machine learning and early neural networks in capturing long-term dependencies and processing high-dimensional data, recent years have seen the widespread application of deep learning and pre-trained models in health intervention decision-making for aviation students. Long Short-Term Memory (LSTM) networks (Zhang et al., 2024a) and Transformer models (Golchha et al., 2024) based on attention mechanisms have significantly enhanced the ability to process complex time series data through deeper network structures and advanced mechanism designs. LSTM, by introducing memory cells, effectively mitigates the vanishing gradient problem and captures long-term dependencies (Kurniawan et al., 2020). The Transformer model, through its attention mechanism, flexibly focuses on important parts of the time series data, making it more adaptable. Additionally, the introduction of pre-trained models, such as BERT (Ouchene and Bessou, 2022) and GPT (Gruver et al., 2024), allows temporal prediction to leverage large-scale pre-training data for more accurate fine-tuning, thereby improving model generalization and prediction accuracy. However, these deep learning methods often require substantial computational resources and data support, leading to higher training costs, and they still face challenges related to interpretability in practical applications. Despite these challenges, the application of deep learning and pre-trained models marks a significant advancement in temporal prediction for health intervention decision-making in aviation students, providing stronger support for aviation safety.

To address the aforementioned challenges, particularly the limitations in capturing long-term dependencies and handling complex, multivariate EEG data in health intervention decision-making for aviation students, we propose our method: PilotCareTrans Net. PilotCareTrans Net is an improved Transformer model designed to enhance the accuracy and efficiency of predicting health interventions based on EEG data in aviation students. This model leverages advanced features such as dynamic attention mechanisms, temporal convolutional layers, and multi-scale feature integration, making it particularly adept at handling the intricate temporal dynamics and non-linear relationships inherent in EEG signals. By building on the strengths of existing models and addressing their weaknesses, PilotCareTrans Net offers a robust solution that not only improves prediction accuracy but also ensures timely and effective health interventions, thereby contributing to enhanced safety and performance in aviation training.

- PilotCareTrans Net introduces a dynamic attention mechanism tailored to EEG data, enabling the model to selectively focus on critical temporal features.
- The model is designed for high efficiency and adaptability across various scenarios, offering robust performance in different health monitoring tasks, making it highly versatile and suitable for real-time applications.
- Comprehensive evaluations reveal that PilotCareTrans Net achieves superior performance over leading models, particularly in critical metrics, demonstrating its practical effectiveness in aviation training environments.

## 2 Related work

### 2.1 Transformer models

Transformer models, initially introduced for natural language processing (NLP), have quickly demonstrated their versatility across a wide range of domains due to their powerful attention mechanisms and ability to process data in parallel. Unlike traditional sequential models such as Recurrent Neural Networks (RNNs) (Zhang D. et al., 2024) and Long Short-Term Memory (LSTM) networks, Transformers do not rely on processing data sequentially. This allows them to capture long-range dependencies and complex relationships in data more efficiently, which is especially useful for tasks requiring a broader understanding of contextual information. In the field of computer vision, Vision Transformers (ViT) have revolutionized image classification by treating images as sequences of patches and using the self-attention mechanism. This approach has enabled ViT models to surpass the performance of traditional Convolutional Neural Networks (CNNs) in many image classification tasks (Althamary et al., 2024). In the realm of time series forecasting, Transformer models have been adapted to handle temporal data. By leveraging their ability to model long-term dependencies without the need for recurrent structures, Transformers have shown considerable improvements in prediction accuracy. These advancements have been particularly impactful in applications such as stock price forecasting (Thwal et al., 2023), weather prediction, and health monitoring. The Transformer's capacity to model complex, multi-step dependencies makes it especially suitable for time series tasks that require understanding patterns across extended periods. In health monitoring, particularly in EEG-based analysis, Transformer models have shown promise by analyzing intricate physiological data to predict health outcomes. The scalability and flexibility of Transformers allow them to process large datasets efficiently, making them an ideal choice for domains that require handling high-dimensional, complex data (Hu et al., 2019). Their ability to parallelize computations also contributes to faster processing, which is crucial in real-time monitoring systems. Overall, Transformers have redefined data processing across various fields, offering superior performance in tasks involving complex temporal and spatial relationships.

### 2.2 Time series health prediction

The field of time series health prediction has seen substantial advancements over the past few decades, evolving from traditional statistical models to more sophisticated deep learning techniques. In the early stages, methods like ARIMA (Lu et al., 2024) and exponential smoothing were commonly used to predict health-related time series data, such as heart rate or blood pressure. These models were favored for their simplicity, ease of interpretation, and effectiveness in capturing short-term trends in the data (Wang et al., 2024). However, they faced significant limitations in handling the non-linear and complex nature of physiological signals, which are often influenced by a wide range of interacting factors and external variables. With the rise of machine learning, more advanced methods were introduced to tackle these complexities. Techniques such as Support Vector Machines (SVM) and Decision Trees began to be applied to health prediction tasks, offering greater flexibility by modeling non-linear relationships within the data (Pierre et al., 2023). These machine learning methods marked a significant improvement over traditional approaches, but they still lacked the ability to fully exploit the temporal dependencies inherent in time series data. The introduction of early neural network models, particularly Recurrent Neural Networks (RNNs) and Long Short-Term Memory (LSTM) networks, brought further advancements in time series health prediction. These models were designed to capture temporal dependencies, making them well-suited for sequential health data such as EEG readings or continuous heart rate monitoring. By maintaining a form of memory over time, RNNs and LSTMs were able to model long-term dependencies more effectively than traditional statistical or machine learning models. More recently, the emergence of Transformer models has brought about a new era in time series health prediction. Unlike recurrent architectures, Transformers can model long-range dependencies and complex patterns without being constrained by the limitations of sequential processing. This has made Transformers particularly effective in predicting various health outcomes by capturing intricate temporal dynamics within large-scale physiological datasets. Their ability to parallelize computations and handle large amounts of data has been a significant step forward in improving the accuracy and scalability of health predictions. Hybrid approaches have also gained attention in recent years, such as the combination of ARIMA with LSTM models, to enhance predictive performance. These hybrid models leverage the strengths of both statistical and deep learning methods, with ARIMA capturing linear trends and LSTM modeling the non-linear temporal relationships (Abgeena and Garg, 2023). These developments reflect a broader trend toward integrating traditional and modern techniques to better address the challenges of health prediction in increasingly complex and high-dimensional datasets.

### 2.3 EEG signals in health monitoring

Electroencephalogram (EEG) signals have become an essential tool in health monitoring, offering crucial insights into the brain's electrical activity, which is vital for diagnosing and predicting various neurological and psychological conditions. EEG signals are particularly valuable in monitoring cognitive states, detecting seizures, and evaluating sleep disorders. Traditionally, the analysis of EEG signals relied on manual feature extraction and conventional statistical methods. While these methods provided some degree of utility, they were limited in their ability to handle the high-dimensional, complex, and often noisy nature of EEG data. This limitation hindered the accuracy and robustness of early EEG-based health assessments. The advent of machine learning marked a significant turning point in EEG signal analysis. More sophisticated models, such as Support Vector Machines (SVM) (Dong et al., 2024) and Neural Networks, began to be employed for automatic feature extraction and classification of EEG signals. These approaches reduced the need for manual intervention, offering improved accuracy and efficiency in detecting and predicting various health conditions, including epilepsy and cognitive disorders (Singh et al., 2023). By modeling the non-linear relationships in EEG data, these machine learning models allowed for more effective utilization of the rich information embedded in EEG signals. In recent years, deep learning techniques have further revolutionized EEG analysis. Particularly, Convolutional Neural Networks (CNNs) (Zhang et al., 2024b) and Transformer models (Li, 2024) have shown great potential in learning relevant features directly from raw EEG data, thus eliminating the need for manual preprocessing and feature engineering. CNNs, known for their ability to capture spatial features, have demonstrated significant effectiveness in EEG-based applications, such as seizure detection, emotion recognition, and sleep disorder classification. The success of CNNs lies in their ability to learn hierarchical representations of EEG data, making them well-suited for handling its complexity. More recently, Transformer models have introduced a new paradigm in EEG analysis. By utilizing self-attention mechanisms, Transformers are capable of capturing long-range dependencies and relationships within EEG signals, leading to more accurate and robust predictions (Hu et al., 2019). These models have been particularly impactful in tasks requiring the modeling of temporal dynamics and interactions across multiple time scales. As a result, the use of Transformers in EEG analysis has significantly enhanced the performance of health monitoring systems. These advancements in EEG signal analysis, driven by machine learning and deep learning technologies, have solidified EEG as a powerful tool for non-invasive, real-time assessment of brain activity in both clinical and research environments. The combination of advanced models and EEG data continues to push the boundaries of health monitoring, enabling earlier diagnosis and more precise treatment of neurological conditions.

## 3 Methodology

### 3.1 Overview

The Transformer architecture has revolutionized the development of sophisticated models, particularly in areas like text processing and visual data analysis, due to its capacity to model complex relationships and dependencies. In this study, we introduce PilotCareTrans Net, a novel Transformer-based model designed to facilitate decision-making in health interventions for aviation students, using EEG data as the primary input. This model leverages the unique strengths of the Transformer architecture, notably its powerful self-attention mechanism, which enables it to capture intricate temporal relationships across multivariate time series data. These capabilities are crucial for unifying diverse EEG signal modalities into a cohesive and integrated system, allowing for more precise and informed decision-making. EEG data, given its high-dimensional and time-sensitive nature, is ideal for capturing physiological states relevant to cognitive performance, stress levels, and overall mental health. In aviation, where safety and performance are paramount, timely health interventions can be critical to maintaining the well-being of pilots and aviation students during both training and real-world operations. PilotCareTrans Net seeks to address this need by providing a robust, data-driven approach to monitoring and predicting health conditions based on EEG signals. By doing so, the model supports the identification of potential health issues, such as fatigue, cognitive overload, or stress, that could impair the performance of aviation students and, ultimately, compromise safety.

**Figure 1:**
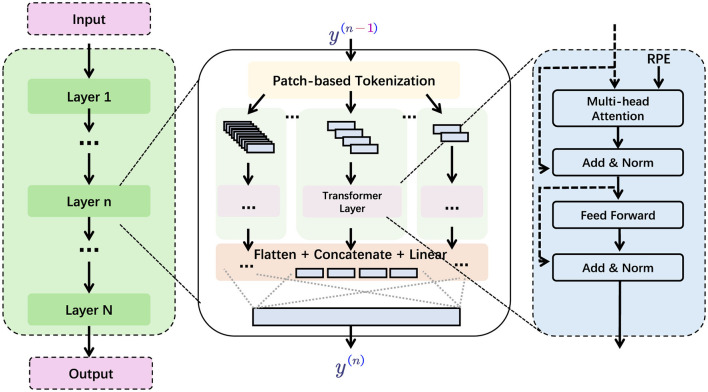
The structure of PilotCareTrans Net. The input is processed through multiple layers (Layer 1 to Layer N), then through the Transformer layer through patch-based segmentation, and finally the output is obtained through flattening, concatenation and linear layers.

To construct PilotCareTrans Net (in Figure 1), we built on established methodologies in time series analysis, drawing from advancements in both EEG signal processing and Transformer-based architectures. We adapted Vision Transformers, which have already demonstrated considerable success in handling visual data, to process multivariate EEG time series. The model restructures the EEG data into a Transformer-compatible format by segmenting the signals into smaller, interpretable units, akin to how Vision Transformers handle images as sequences of patches. This restructuring allows the model to capture both short-term fluctuations and long-term dependencies within the EEG signals, thereby enhancing its ability to make accurate health-related predictions. One of the key innovations of PilotCareTrans Net is its ability to unify different modalities of EEG signals, which typically contain a wide range of information related to brain activity, cognitive function, and mental health. By integrating these signals into a single model, PilotCareTrans Net not only improves the clarity of the predictions but also increases the system's effectiveness in real-world applications. This is particularly beneficial in aviation education, where the ability to assess and intervene based on the mental and physical well-being of students can directly impact their learning outcomes, performance, and safety.

### 3.2 Preliminaries

To tackle the challenge of making health intervention decisions for aviation students using EEG data, it is critical to first establish a mathematical framework. Let *X* ∈ ℝ^*N*×*T*×*C*^ represent the multivariate EEG time series, where *N* indicates the total number of samples, *T* refers to the number of time steps, and *C* stands for the EEG channels. Each sample $X_{i}\in {\mathbb{R}}^{T\times C}$ comprises *T* time steps of *C*-channel EEG signals. The primary aim is to construct a model that can reliably predict the necessary health intervention for a student based on the EEG time series *X*_*i*_. This objective can be formalized as a classification problem, where the goal is to learn a mapping function *f*:ℝ^*T*×*C*^ → ℝ^*K*^, with *K* representing the number of possible health intervention outcomes. The function *f*(*X*_*i*_; θ) is parameterized by θ and outputs the likelihood distribution across the *K* classes. Given *N* training instances ${\left\{X_{i},y_{i}\right\}}_{i=1}^{N}$, where *y*_*i*_ ∈ {1, 2, …, *K*} denotes the actual health intervention category for the *i*-th sample, the learning process involves optimizing a loss function L(θ) that quantifies the difference between the predicted and actual labels. A common choice for L(θ) is the cross-entropy loss, defined as:


(1) $$\begin{array}{l} L\left(\theta \right)=-\frac{1}{N}\overset{N}{\underset{i=1}{\sum }}\overset{K}{\underset{k=1}{\sum }}𝕀\left(y_{i}=k\right)\log p_{k}\left(X_{i};\theta \right), \end{array}$$

where *p*_*k*_(*X*_*i*_; θ) gives the predicted likelihood for class *k* for sample *X*_*i*_, and 𝕀(·) is an indicator function.

In the context of EEG analysis, capturing the temporal dynamics across various channels is crucial. Therefore, the input time series *X*_*i*_ undergoes a preprocessing step to extract meaningful temporal features. Let $Z_{i}\in {\mathbb{R}}^{T^{\prime }\times D}$ represent the transformed feature matrix, where *T*′ ≤ *T* is the reduced number of time steps after preprocessing, and *D* is the dimensionality of the extracted features. This transformation may involve operations like downsampling, filtering, or feature extraction using domain-specific methods.

The preprocessed data **Z**_*i*_ is then fed into a Transformer-based encoder, which captures the temporal dependencies and interactions among the features. Each layer in the encoder can be expressed as a function *h*^(*l*)^:ℝ^*T*′×*D*^ → ℝ^*T*′×*D*^, where *l* denotes the layer index. The output of the *L*-th layer of the encoder, ${\text{H}}_{i}^{\left(L\right)}\in {\mathbb{R}}^{T^{\prime }\times D}$, serves as the input to the decision-making module.

The decision-making module aggregates the information across all time steps to produce the final prediction. This can be achieved using various pooling strategies or an additional attention mechanism. Let *o*:ℝ^*T*′×*D*^ → ℝ^*D*′^ represent this aggregation function, which outputs a fixed-size representation ${\text{v}}_{i}\in {\mathbb{R}}^{D^{\prime }}$ for each sample. The final classification is performed by a fully connected layer followed by a softmax function, mapping **v**_*i*_ to the predicted probability distribution ${ŷ}_{i}\in {\mathbb{R}}^{K}$:


(2) $$\begin{array}{l} {ŷ}_{i}=\text{softmax}\left(\text{W}{\text{v}}_{i}+\text{b}\right), \end{array}$$

where **W** ∈ ℝ^*K*×*D*′^ and **b** ∈ ℝ^*K*^ are the parameters of the output layer.

The EEG time series data are divided into non-overlapping time-windows of 10 s, with a sliding window approach used to extract segments from the continuous EEG signal. The step size between consecutive windows is 2 s, which ensures both high temporal resolution and sufficient data for analysis. This choice is based on the need to balance temporal accuracy with computational efficiency.

The choice of transformation operation is influenced by factors such as the signal-to-noise ratio, frequency components, and the nature of the analysis, whether it is focused on noise reduction, feature extraction, or dimensionality reduction. For example, when processing EEG data containing high-frequency noise, bandpass filtering or notch filtering are applied to remove unwanted frequencies and retain relevant signals. In datasets with a large number of channels or high-dimensional data, dimensionality reduction techniques such as principal component analysis (PCA) or independent component analysis (ICA) are used to reduce the dimensionality of the data and retain the most important features. The manuscript stipulates that the choice of preprocessing operations is tailored to the specific characteristics of each dataset. In some cases, temporal smoothing or artifact removal techniques can be applied, while in other datasets, frequency domain transforms such as fast Fourier transform (FFT) or wavelet transform can be used. This ensures that the preprocessing operations are appropriate for the dataset, allowing the model to extract the most relevant information while maintaining data integrity.

### 3.3 Adaptive temporal encoder network

The central innovation of PilotCareTrans Net lies in its Adaptive Temporal Encoder Network (ATEN), a specialized module designed to enhance the Transformer's capacity to handle the unique characteristics of EEG data. EEG signals are known for their temporal complexity and high variability across different individuals and conditions. The ATEN is built upon the standard Transformer encoder but incorporates dynamic attention mechanisms and temporal convolutional layers, which are tailored for processing EEG signals. The architecture of ATEN can be described as follows.

#### 3.3.1 Input representation

the raw EEG data, represented by **X** ∈ ℝ^*N*×*T*×*C*^, undergoes a transformation into a feature matrix **Z** ∈ ℝ^*T*′×*D*^ using a preprocessing function *g*(**X**). This preprocessing function includes operations such as temporal filtering and feature extraction, resulting in a reduced representation that retains the most critical temporal patterns.

#### 3.3.2 Dynamic attention mechanism

To effectively capture the varying importance of EEG signals over time, the Adaptive Temporal EEG Network (ATEN) employs a dynamic attention mechanism. This is a significant improvement over the fixed self-attention mechanism found in traditional Transformers. In contrast to the standard self-attention, where attention weights remain fixed once learned, the dynamic attention in ATEN adapts its focus continuously based on the evolving input features at each time step. This makes the model more responsive to the unique characteristics of the temporal data, particularly in the context of EEG signals, which exhibit varying patterns of relevance over time.

At each time step *t*, the attention weights ${\text{A}}_{t}\in {\mathbb{R}}^{T^{\prime }\times T^{\prime }}$ are dynamically computed as follows:


(3) $$\begin{array}{l} {\text{A}}_{t}=\text{softmax}\left(\frac{{\text{Q}}_{t}{\text{K}}_{t}^{⊤}}{\sqrt{D}}\right), \end{array}$$

where **Q**_*t*_ represents the query vector at time step *t*, and **K**_*t*_ represents the key vectors corresponding to the sequence data. These are computed as:


(4) $$\begin{array}{l} {\text{Q}}_{t}={\text{W}}_{Q}{\text{z}}_{t},\;{\text{K}}_{t}={\text{W}}_{K}\text{Z}, \end{array}$$

where ${\text{z}}_{t}\in {\mathbb{R}}^{D}$ is the input feature vector at time step *t*, **Z** ∈ ℝ^*T*′×*D*^ is the matrix of all input feature vectors over time, ${\text{W}}_{Q},{\text{W}}_{K}\in {\mathbb{R}}^{D\times D}$ are learnable weight matrices for the query and key projections, respectively, *D* is the dimensionality of the hidden representations, and **A**_*t*_ are the attention weights at time *t*, which reflect the model's focus on different time steps based on the temporal context.

The softmax function ensures that the attention weights are normalized, meaning they sum to 1 across the time steps, and it accentuates the most important relationships between different parts of the sequence:


(5) $$\begin{array}{l} \text{softmax}\left(x_{i}\right)=\frac{\exp\left(x_{i}\right)}{\sum_{j=1}^{T^{\prime }}\exp\left(x_{j}\right)}, \end{array}$$

where *x*_*i*_ corresponds to each element in the attention score matrix.

This dynamic formulation allows the attention weights to shift based on the input features, which is particularly useful when dealing with EEG data that often has fluctuating periods of importance. For example, certain brainwave activities during critical events, such as the onset of a seizure or a state of deep sleep, may be more relevant for prediction at certain time points. The dynamic attention mechanism ensures that the model can increase its focus on such crucial moments by adjusting the weights **A**_*t*_ accordingly.

The process for obtaining the value vector **V**_*t*_, which represents the output of the attention mechanism, is similar to traditional self-attention. It is computed as:


(6) $$\begin{array}{l} {\text{V}}_{t}={\text{A}}_{t}{\text{W}}_{V}\text{Z}, \end{array}$$

where ${\text{W}}_{V}\in {\mathbb{R}}^{D\times D}$ is another learnable weight matrix, and **Z** is again the input feature matrix. The output **V**_*t*_ aggregates information from different time steps based on the attention weights, dynamically emphasizing the most relevant periods.

Furthermore, the dynamic attention mechanism can be enhanced by incorporating positional encoding **P** ∈ ℝ^*T*′×*D*^, which allows the model to retain information about the relative order of time steps, a critical aspect of time series data. Thus, the query and key vectors can be reformulated as:


(7) $$\begin{array}{l} {\text{Q}}_{t}={\text{W}}_{Q}\left({\text{z}}_{t}+{\text{p}}_{t}\right),\;{\text{K}}_{t}={\text{W}}_{K}\left(\text{Z}+\text{P}\right), \end{array}$$

where ${\text{p}}_{t}\in {\mathbb{R}}^{D}$ is the positional encoding for time step *t*. This allows the model to capture both the content of the EEG signals and their temporal ordering.

#### 3.3.3 Temporal convolutional layers

In addition to the dynamic attention mechanism, the Adaptive Temporal EEG Network (ATEN) incorporates temporal convolutional layers that operate on the feature matrix **Z**. These convolutional layers are designed to capture local temporal dependencies within the EEG signals, while also reducing the impact of noise, which is a common issue in EEG data due to its high variability and susceptibility to external interference. The temporal convolutional layers allow the model to extract features that are locally significant in time, such as sharp transitions or rhythmic oscillations in brainwave patterns.

The output of a temporal convolutional layer at time step *t* is computed as:


(8) $$\begin{array}{l} {\text{C}}_{t}=\sigma \left({\text{W}}_{C}*\text{Z}\right), \end{array}$$

where * denotes the convolution operation applied over the time dimension, ${\text{W}}_{C}\in {\mathbb{R}}^{k\times D\times D}$ is the convolution kernel (or filter) with a receptive field of size *k*, and σ(·) is a non-linear activation function, commonly chosen as Rectified Linear Unit (ReLU) or another suitable function. In this context, *k* represents the kernel size, controlling how many time steps are considered simultaneously during the convolution operation. The activation function σ(·) introduces non-linearity into the model, enabling it to capture more complex temporal patterns and relationships that may not be detectable through linear operations alone.

The convolution operation, defined by:


(9) $$\begin{array}{l} {\left({\text{W}}_{C}*\text{Z}\right)}_{t}=\overset{k}{\underset{i=1}{\sum }}{\text{W}}_{C}\left(i\right)\cdot {\text{Z}}_{t-i+1:t-i+k}, \end{array}$$

aggregates information across *k* consecutive time steps of the input feature matrix **Z**, effectively capturing local patterns within that time window. By applying multiple convolutional layers, ATEN builds hierarchical representations of the EEG data, allowing the model to understand both low-level temporal features (e.g., short oscillations or spikes) and higher-level abstractions (e.g., complex brainwave patterns over longer durations).

#### 3.3.4 Residual connections and layer normalization

To ensure efficient training of deep neural networks, residual connections and layer normalization are essential components of the ATEN architecture. Residual connections are employed to mitigate the problem of vanishing or exploding gradients, which can occur as the network depth increases. In deep networks, gradients can become extremely small (vanishing gradient problem) or grow too large (exploding gradient problem), making it difficult to train the model effectively. By incorporating residual connections, ATEN helps maintain a smooth gradient flow throughout the network, ensuring that important information is propagated forward and backward during training.

The output of each attention layer is added to its corresponding input, forming a residual black. Mathematically, this is expressed as:


(10) $$\begin{array}{l} {\text{H}}_{t}^{\left(l+1\right)}={\text{H}}_{t}^{\left(l\right)}+\text{Attention}\left({\text{H}}_{t}^{\left(l\right)}\right), \end{array}$$

where ${\text{H}}_{t}^{\left(l\right)}$ represents the input to the attention layer at level *l*, and ${\text{H}}_{t}^{\left(l+1\right)}$ is the output after the residual connection has been applied. The use of residual connections allows the network to bypass certain layers if necessary, which can help prevent the degradation of performance as the network grows deeper. It also ensures that the model can learn both shallow and deep representations simultaneously.

To further stabilize training, layer normalization is applied to the output of the residual black. This operation normalizes the activations at each layer, improving the stability of the training process by ensuring that the inputs to each layer maintain a consistent scale and distribution. The full process is described as:


(11) $$\begin{array}{l} {\text{H}}_{t}^{\left(l+1\right)}=\text{LayerNorm}\left({\text{H}}_{t}^{\left(l\right)}+\text{Attention}\left({\text{H}}_{t}^{\left(l\right)}\right)\right), \end{array}$$

where LayerNorm(·) normalizes the output of the residual block by adjusting the mean and variance of the activations, ensuring they are more suitable for subsequent layers. Layer normalization is particularly useful in time series data, where fluctuations in scale or distribution between different time steps can introduce instability in training. By applying layer normalization, ATEN maintains a consistent gradient flow and ensures that the learning process remains stable across layers, even in very deep architectures.

#### 3.3.5 Multi-scale feature integration

EEG signals exhibit complex temporal dynamics that span multiple time scales, making it essential for models like ATEN to capture both short-term and long-term dependencies. To address this, ATEN incorporates a multi-scale feature integration mechanism. This mechanism ensures that features extracted at different temporal resolutions contribute to a more comprehensive and robust representation of the EEG data.

Let **F**_1_, **F**_2_, …, **F**_*M*_ denote the features extracted from *M* different temporal scales, where each ${\text{F}}_{i}\in {\mathbb{R}}^{D_{i}^{\prime }}$ corresponds to a specific time scale *i*. These features, representing different aspects of the EEG signals, are concatenated to form a unified feature vector. The concatenation operation aggregates the information across all scales into a single, enriched representation that captures both fine-grained and coarse-grained patterns.

The combined feature vector is then passed through a fully connected layer to further refine the integrated features and produce a compact representation that is suitable for the subsequent decision-making process. Mathematically, the multi-scale feature integration process is described as:


(12) $$\begin{array}{l} {\text{F}}_{\text{int}}=\text{FC}\left(\text{Concat}\left({\text{F}}_{1},{\text{F}}_{2},\ldots ,{\text{F}}_{M}\right)\right), \end{array}$$

where Concat(·) represents the concatenation operation over the multi-scale feature vectors **F**_1_, **F**_2_, …, **F**_*M*_, and FC(·) is a fully connected layer that projects the concatenated features into a lower-dimensional space. This integrated feature vector, denoted as **F**_int_, encapsulates the rich temporal information present in the EEG data across different time scales, allowing the model to effectively capture both short-term fluctuations and long-term trends.

By combining features from multiple temporal scales, ATEN can better handle the varying patterns found in EEG data, such as brief spikes or sustained oscillations. This multi-scale integration ensures that the model is sensitive to both the immediate and prolonged changes in brain activity, which is critical for accurate prediction of health interventions, especially in dynamic environments like aviation training.

The final stage of the ATEN architecture is the prediction head, which maps the integrated feature vector **F**_int_ to the output space, providing the final predictions for the health intervention decision-making task. After processing the multi-scale integrated features, the prediction head applies a fully connected layer to project **F**_int_ into the output space, followed by a softmax activation function to produce a probability distribution over the possible health intervention classes.

The prediction head can be mathematically formulated as:


(13) $$\begin{array}{l} {ŷ}_{i}=\text{softmax}\left({\text{W}}_{o}{\text{F}}_{\text{int}}+{\text{b}}_{o}\right), \end{array}$$

where: ${ŷ}_{i}\in {\mathbb{R}}^{K}$ is the predicted probability vector for each of the *K* health intervention classes, ${\text{W}}_{o}\in {\mathbb{R}}^{K\times D^{\prime }}$ is the weight matrix of the output layer, where *D*′ is the dimensionality of the integrated feature vector **F**_int_, ${\text{b}}_{o}\in {\mathbb{R}}^{K}$ is the bias term of the output layer.

The softmax function normalizes the output into a probability distribution:


(14) $$\begin{array}{l} \text{softmax}\left(z_{i}\right)=\frac{\exp\left(z_{i}\right)}{\sum_{j=1}^{K}\exp\left(z_{j}\right)}, \end{array}$$

where *z*_*i*_ represents the unnormalized logits (i.e., **W**_*o*_**F**_int_ + **b**_*o*_) for class *i*. The softmax function ensures that the output probabilities sum to 1, which is necessary for multi-class classification tasks. In the context of ATEN, this enables the model to output a probability for each health intervention class, providing a clear decision on which intervention is most appropriate based on the EEG data.

The integration of the multi-scale feature mechanism and the prediction head forms a key component of ATEN's architecture. By capturing both short-term and long-term dependencies through multi-scale feature extraction, and making accurate predictions through the softmax-based classification in the prediction head, ATEN delivers robust and reliable decisions for health interventions. This design is particularly advantageous in contexts like aviation training, where timely and precise health assessments are critical for ensuring both safety and performance. The Adaptive Temporal Encoder Network, with its tailored components, significantly enhances the ability of PilotCareTrans Net to interpret complex EEG data, making it a powerful tool for decision-making in health interventions for aviation students.

### 3.4 Incorporation of domain-specific strategies

The effectiveness of PilotCareTrans Net is further enhanced by the integration of domain-specific strategies that are tailored to the unique characteristics of EEG data and the specific requirements of health intervention decision-making in aviation students. These strategies involve the incorporation of prior knowledge about EEG signal processing, the adaptation of model components to reflect the physiological and cognitive aspects of aviation students, and the use of specialized training procedures to improve model robustness and interpretability.

#### 3.4.1 omain-specific feature engineering

One of the key strategies involves the engineering of features that are particularly relevant to the aviation context (Gauba et al., 2017). EEG signals are known to contain information about various cognitive states, such as alertness, stress, and fatigue, which are critical for aviation performance. To capture these states, we incorporate features such as power spectral density (PSD) in specific frequency bands (e.g., alpha, beta, theta, and delta), as well as event-related potentials (ERPs) that are known to correlate with cognitive and emotional responses (Zhang Z. et al., 2024). These features are computed from the raw EEG data during the preprocessing stage and are included in the feature matrix **Z** before it is input into the Adaptive Temporal Encoder Network.

#### 3.4.2 Physiological and cognitive constraints

To align the model's predictions with physiological and cognitive principles, we impose constraints on the learned representations and predictions (Kumar et al., 2019). For instance, the model is trained to recognize patterns in the EEG data that are consistent with known cognitive states under various conditions, such as high cognitive load or low alertness. These constraints are implemented by incorporating regularization terms into the loss function, which penalize predictions that deviate from expected physiological responses. The regularization term R(θ) can be formulated as:


(15) $$\begin{array}{l} R\left(\theta \right)=\lambda \overset{N}{\underset{i=1}{\sum }}\overset{C}{\underset{j=1}{\sum }}|{ŷ}_{i}^{\left(j\right)}-{ỹ}_{i}^{\left(j\right)}|, \end{array}$$

where ${ŷ}_{i}^{\left(j\right)}$ is the predicted probability of class *j* for sample *i*, ${ỹ}_{i}^{\left(j\right)}$ represents the expected probability distribution based on cognitive state constraints, and λ is a hyperparameter that controls the strength of the regularization.

#### 3.4.3 Attention to temporal dynamics

The temporal nature of EEG signals necessitates a model that can effectively capture both short-term fluctuations and long-term trends in the data (Prasad et al., 2018). To this end, we employ a hierarchical attention mechanism that assigns varying levels of importance to different temporal segments of the EEG data. This mechanism enables the model to focus on critical moments, such as periods of high cognitive demand or physiological stress, which are particularly relevant for health intervention. The hierarchical attention weights are computed as:


(16) $$\begin{array}{l} {\text{A}}_{\text{hier}}=\text{softmax}\left(\frac{{\text{Q}}_{\text{hier}}{\text{K}}_{\text{hier}}^{⊤}}{\sqrt{D_{\text{hier}}}}\right), \end{array}$$

where **Q**_hier_ and **K**_hier_ are the query and key matrices at the hierarchical level, and *D*_hier_ is the dimensionality of the hierarchical attention space.

#### 3.4.4 Robust training procedures

Given the variability and noise inherent in EEG data, robust training procedures are essential for ensuring that the model generalizes well to unseen data (Dash et al., 2024). We incorporate several techniques to enhance the robustness of PilotCareTrans Net, including data augmentation, dropout, and cyclic learning rates. Data augmentation is performed by applying perturbations such as noise addition, time-warping, and channel dropout to the EEG signals, which helps the model become more resilient to variations in the data. The cyclic learning rate schedule is used to adaptively adjust the learning rate during training, encouraging the model to explore different regions of the parameter space and avoid local minima. The cyclic learning rate is defined as:


(17) $$\begin{array}{l} \eta \left(t\right)=\eta_{\text{min}}+\frac{1}{2}\left(\eta_{\text{max}}-\eta_{\text{min}}\right)\left(1+\cos\left(\frac{t\pi }{T_{\text{cycle}}}\right)\right), \end{array}$$

where η(*t*) is the learning rate at time step *t*, η_min_ and η_max_ are the minimum and maximum learning rates, respectively, and *T*_cycle_ is the length of one learning rate cycle.

## 4 Experiment

### 4.1 Datasets

The experimental evaluation of PilotCareTrans Net was conducted using four diverse EEG datasets, each providing unique challenges and characteristics relevant to health intervention decision-making. The MODA Dataset (Lacourse et al., 2020) is a comprehensive collection that captures EEG signals across various cognitive tasks, offering a broad spectrum of physiological states. The STEW Dataset (Lim et al., 2018), known for its detailed annotation of stress levels, provides a rich resource for studying the impact of stress on cognitive performance in aviation students. The SJTU Emotion EEG Dataset (Zheng and Lu, 2015; Duan et al., 2013), focused on emotion recognition, adds another layer of complexity by emphasizing the detection of subtle emotional states from EEG signals. Lastly, the Sleep-EDF Dataset (Goldberger et al., 2000), which includes recordings of EEG during sleep, allows for the exploration of sleep-related cognitive states and their implications for aviation safety. These datasets collectively represent a wide array of scenarios, from awake cognitive states to sleep conditions, making them ideal for evaluating the robustness and versatility of the proposed model.

The experiments in this study utilized four publicly available EEG datasets, which are summarized in Table 1. The MODA dataset includes EEG recordings from 25 healthy adults with a sampling rate of 256 Hz. The dataset is focused on cognitive workload assessment and sleep spindle detection. Preprocessing involved applying a bandpass filter in the range of (0.5–50 Hz) and down-sampling the signals to 100 Hz to ensure consistency. The STEW dataset consists of EEG recordings from 15 participants, collected at a sampling rate of 500 Hz, with experimental tasks centered on mental workload assessment. Preprocessing for this dataset included a bandpass filter in the range of (1–50 Hz) and down-sampling to 100 Hz. The SEED dataset contains EEG data collected from 15 participants at 200 Hz during emotion recognition tasks, which required classifying emotional states such as happy, neutral, and sad. For this dataset, preprocessing steps included a bandpass filter in the range of (0.5–50 Hz) and down-sampling to 100 Hz. The Sleep-EDF dataset includes EEG signals from 20 participants collected at 100 Hz, focused on sleep stage classification tasks. Preprocessing for this dataset required only a bandpass filter in the range of (0.5–30 Hz), with no additional down-sampling applied.

**Table 1:** Summary of EEG datasets and experimental settings.

**Dataset**	**Subjects**	**Sampling rate (Hz)**	**Tasks/conditions**	**Preprocessing steps**	**References**
MODA	25 healthy adults	256	Cognitive workload and sleep spindles	Band-pass filtering (0.5–50 Hz), down-sampling to 100 Hz	Lacourse et al. (2020)
STEW	15 participants	500	Mental workload tasks	Band-pass filtering (1–50 Hz), down-sampling to 100 Hz	Lim et al. (2018)
SEED (SJTU)	15 participants	200	Emotion recognition tasks (happy, neutral, sad)	Band-pass filtering (0.5–50 Hz), down-sampling to 100 Hz	Zheng and Lu (2015)
Sleep-EDF	20 participants	100	Sleep stage classification	No further down-sampling needed, band-pass filtering (0.5–30 Hz)	Goldberger et al. (2000)

In addition to the dataset-specific preprocessing, all datasets underwent consistent steps to enhance signal quality and ensure compatibility with the model. EEG signals were normalized using *z*-score normalization to eliminate amplitude differences between individuals. Signals were divided into 10-s non-overlapping time windows, with each window containing 1,000 time points to maintain a uniform sample length. Further feature extraction was applied to enhance the relevance of the data for classification tasks. A bandpass filter was applied to remove low-frequency drift and high-frequency noise, such as muscle artifacts, ensuring the retention of critical signal features. The power spectral density was estimated using the Welch method with a 50% overlap between segments and frequency bands of interest, such as delta, theta, alpha, and beta, were extracted. Event-related potentials were also computed by averaging EEG signals within a time window of (–200 ms, +800 ms) relative to specific target events, capturing the temporal dynamics associated with cognitive processing. These steps ensured that the data was clean, informative, and ready for classification using the Transformer-based architecture. All datasets were evaluated independently to avoid biases caused by merging data from different sources. A 10-fold cross-validation strategy was implemented to assess the model's generalization ability, where the test set consisted exclusively of unseen participant records. This ensured that the evaluation process focused on the model's capacity to generalize across different individuals rather than learning subject-specific patterns. These carefully designed preprocessing and evaluation procedures allowed the model to effectively leverage the temporal and spectral features of EEG data to classify cognitive states and support health intervention decision-making.

The EEG data are represented as a 3D matrix **X** ∈ ℝ^30×5,000×32^, where *N* = 30 subjects, each providing 5,000 time steps recorded over 32 EEG channels. Each time step represents a sample of 32 EEG channels, and the total number of samples for the dataset is 30 × 5,000 = 150, 000 samples across all subjects. It should be noted that this matrix is provided as an example based on a specific dataset. For the other datasets, the data matrices were prepared according to their respective characteristics, such as the number of subjects, time steps, and EEG channels, as summarized in Table 1. To mitigate the risk of overfitting due to the high dimensionality of the data, several regularization techniques were employed. A dropout rate of 0.5 was applied to all hidden layers to prevent the model from overfitting to the training data. Additionally, training was halted when the validation loss stopped improving for five consecutive epochs. Data augmentation techniques such as random time-shifting and noise addition were also utilized to increase the effective size of the training set and ensure that the model did not learn trivial patterns. These steps helped improve the generalization performance of the model while effectively handling the variability inherent in EEG data.

To enhance the variability and robustness of the model, we apply data augmentation techniques in a cascade manner. First, we introduce random time-shifting to the EEG signals, with each signal being shifted by a random amount of up to ±50 ms along the time axis. This shift simulates small temporal variations in the timing of events and helps the model learn to generalize across slight misalignments without overfitting to precise event timings. In addition to time-shifting, Gaussian noise is added to the signals to simulate environmental noise and recording artifacts. The noise is introduced with a standard deviation of 5% of the original signal amplitude, ensuring that the noise level is realistic without distorting the underlying EEG data. For each dataset, the augmented samples are generated at a ratio of 2:1 relative to the original data, resulting in a total dataset size three times the original. This augmentation level was chosen to balance diversity and computational feasibility while avoiding over-reliance on the original data for manipulations. These augmentations help the model become more resilient to minor signal variations and improve its robustness when exposed to real-world conditions. The augmentation techniques are applied uniformly across all datasets, without dataset-specific selection, meaning that the same augmentation procedures are applied to every dataset in the study. This ensures that the model is trained with a consistent strategy, and the augmented data reflects realistic variations in the EEG signals. Importantly, we designed the augmentation methods to avoid introducing unrealistic distortions that could bias the model or degrade the quality of the data. By preserving the core temporal and spectral characteristics of the EEG signals while increasing variability, these methods allow the model to learn relevant features without memorizing noise or irrelevant patterns.

### 4.2 Experimental setup

In the experimental setup, the dataset was divided into training, validation, and test sets using a stratified sampling technique to ensure that the class distribution in each subset accurately reflects that of the overall dataset. To avoid potential data leakage, a subject-wise split method was adopted, ensuring that all EEG data from a single subject appeared in only one subset: either the training, validation, or test set. This strict division ensures that the model's performance reflects its generalization capability to unseen individuals. Ultimately, the dataset was split into 70% for training, 15% for validation, and 15% for testing. The EEG data underwent preprocessing, including band-pass filtering to remove noise and artifacts, and the extraction of features such as power spectral density and event-related potentials to enhance the model's ability to capture cognitive and emotional states. Additionally, data augmentation techniques, such as random time shifts and noise addition, were applied to improve the model's robustness. The model, PilotCareTrans Net, was implemented using the PyTorch framework and trained on an NVIDIA Tesla V100 GPU with CUDA acceleration. The Adam optimizer was used for training, with an initial learning rate set to 0.001, a common configuration in deep learning that achieves a balance between convergence speed and stability, as validated by preliminary experiments. To further improve training efficiency and performance, a cyclic learning rate strategy was employed, decaying the learning rate by a factor of 0.1 every 10 epochs. The batch size was set to 64, based on validation experiments comparing batch sizes of 32, 64, and 128, and was chosen to balance computational efficiency and memory requirements. The model was trained for a maximum of 50 epochs, with early stopping applied based on validation loss to prevent overfitting. Moreover, a dropout rate of 0.5 was introduced to enhance generalization performance; this value outperformed alternatives of 0.3 and 0.7 in preliminary experiments. The selection of hyperparameters was informed by domain knowledge, standard practices from the literature, and results from initial experimental validation, ensuring the model's robustness and efficiency while adhering to common optimization strategies for EEG time series classification tasks (Algorithm 1).

**Algorithm 1:**
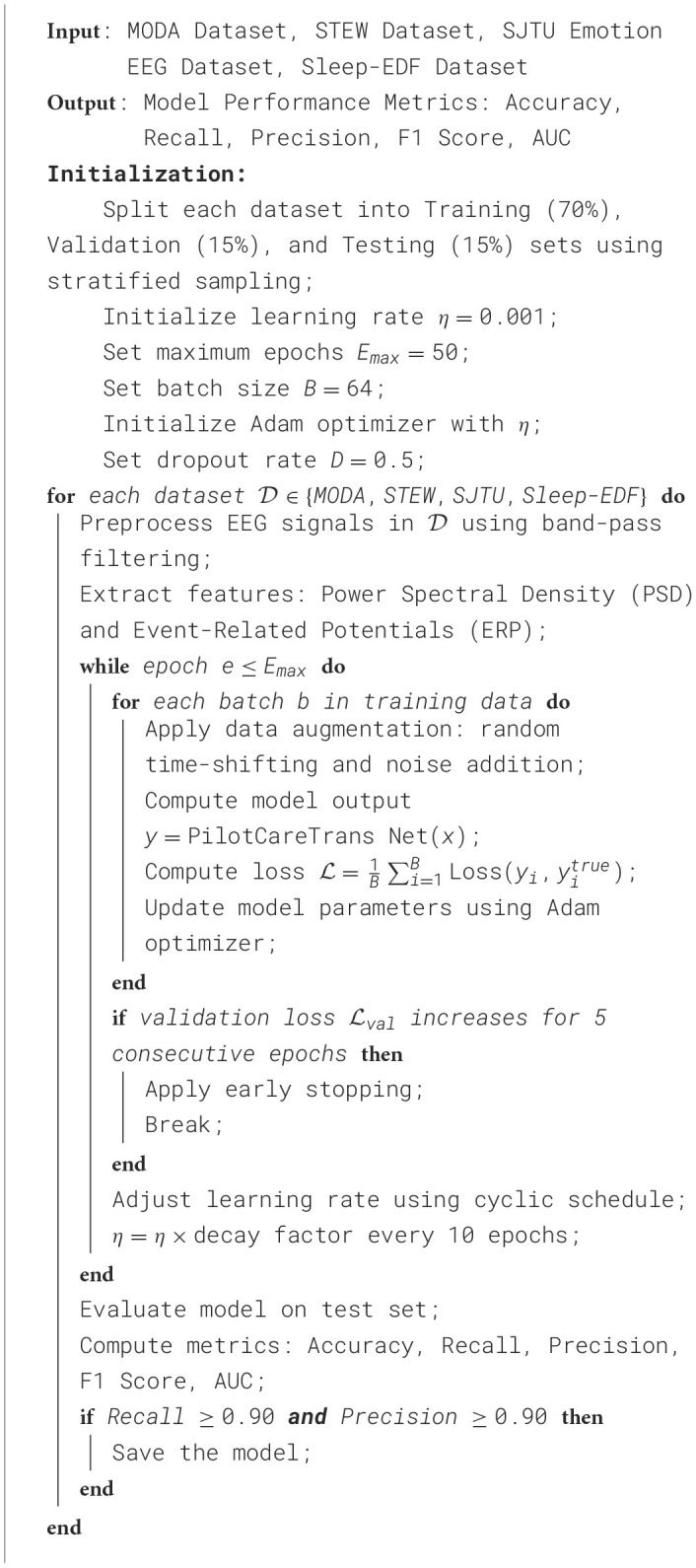
Training and evaluation of PilotCareTrans Net.

### 4.3 Experimental results and analysis

In this section, we focus on the evaluation of our model's performance in predicting health interventions based on EEG data. The core task of this study is to classify cognitive states from EEG signals and map them to appropriate health interventions. These interventions include actions such as rest, cognitive exercises, stress management techniques, and alertness stimulation, all of which are tailored to address specific cognitive states.

The classification task in this paper is to predict appropriate health interventions based on different levels of cognitive states. The classification process is based on specific features extracted from EEG signals, including changes in power spectral density (PSD) in different frequency bands and event-related potential (ERP) characteristics. Cognitive states are divided into high cognitive load (HCL), mental fatigue (MF), stress (S), wakefulness (A) and low wakefulness (LA), and correspond to specific health interventions such as rest, cognitive practice, stress management techniques, and wakefulness stimulation. Table 2 lists the distribution of cognitive states in the sample data in detail, among which the five cognitive states are high cognitive load (23.3%), mental fatigue (20.0%), stress (26.7%), wakefulness (16.7%), and low wakefulness (13.3%). The classification of these states is based on features extracted from EEG signals, such as power spectral density (PSD) and event-related potential (ERP) markers in specific frequency bands, and the classification results correspond one by one to the health intervention strategies defined by experts.

**Table 2:** Data distribution across cognitive states.

**Cognitive state**	**Number of samples**	**Percentage (%)**	**Associated dataset(s)**
High cognitive load (HCL)	3,500	23.3	MODA, STEW
Mental fatigue (MF)	3,000	20.0	MODA, SEED
Stress (S)	4,000	26.7	STEW, SEED
Alertness (A)	2,500	16.7	MODA, Sleep-EDF
Low alertness (LA)	2,000	13.3	Sleep-EDF, MODA
**Total**	**15,000**	**100.0**	All datasets

Bold values are the best values.

Recommendations for health interventions are based on the classification results of these cognitive states, and the true labels of the classifications are defined by experts through a combination of task performance, self-reported stress, and EEG indicators. These definitions are triggered by empirical thresholds that are determined based on expert evaluations of behavioral and physiological data. For example, high cognitive load may be associated with increased activity in a specific frequency band, and mental fatigue may be manifested as a decrease in alpha frequency and an increase in theta frequency. **Table** **3** summarizes in detail the classification conditions of cognitive states, the EEG features used for classification, the relevant health interventions, and the true basis for defining these states. For the selection of different datasets, the applicability of each dataset depends on the characteristics of the EEG data it provides. For example, although the Sleep-EDF dataset is derived from polysomnography, by analyzing features related to wakefulness and sleep stages (such as delta activity and overall EEG rhythm), we can infer some cognitive characteristics related to low wakefulness states. For the use of these datasets, we ensured their relevance to the task of this article through specific feature extraction methods. The relationship between the classification results and the recommendation of interventions is as follows: the algorithm classifies cognitive states, and **Table** **3** provides health intervention suggestions based on these states. Therefore, the recommendation process actually matches cognitive states with predefined health intervention strategies through classification results. This approach ensures a close link between classification and health intervention strategies, while retaining the flexibility for experts to make further decisions based on specific conditions.

The classification task in this study aims to predict appropriate health interventions based on EEG data, which are categorized according to cognitive states. These cognitive states are identified through specific features derived from the EEG signals, such as power spectral density (PSD) in different frequency bands and event-related potentials (ERP). The health interventions correspond to specific cognitive states, and are designed to address cognitive load, fatigue, stress, alertness, and low alertness. Table 3 summarizes the classification conditions (cognitive states), the features used for classification, the associated health interventions, and the ground-truth definitions. The cognitive states considered in the model include high cognitive load (HCL), mental fatigue (MF), stress (S), alertness (A), and low alertness (LA). For each cognitive state, relevant EEG features are extracted, such as changes in alpha, theta, and beta frequency bands, and ERP markers. Based on these features, specific health interventions are recommended, such as rest, cognitive exercises, stress management techniques, and alertness stimulation. The ground-truth for each cognitive state is defined by expert judgment, based on a combination of task performance, self-reported stress, and EEG indicators. The table also illustrates how these interventions are triggered according to predefined thresholds, which are empirically determined and based on expert evaluation of both behavioral and physiological data.

**Table 3:** Classification conditions (CS), health interventions (HI), and ground-truth (GT) definitions.

**CS**	**F**	**HI**	**GT**
HCL	PSD (beta, theta), ERP latency	Rest, cognitive exercise	Triggered by cognitive load threshold (task performance, EEG)
MF	Alpha decrease, PSD (Delta)	Rest, Fatigue monitoring	Based on task time, error rate, and fatigue-related EEG features
S	Theta increase, ERP	Stress management, relaxation techniques	Based on expert judgment (self-reported stress, EEG stress markers)
A	Alpha increase, low beta	Cognitive reinforcement, monitoring	Confirmed by EEG indicators of alertness and low cognitive load
LA	Alpha decrease, low theta/beta	Stimulation, cognitive load management	Expert labels based on self-reported fatigue and EEG features of low alertness

CS, cognitive state; F, feature; HI, health intervention; GT, ground-truth.

HI includes rest, cognitive exercise, stress management, and monitoring.

**Figure 2:**
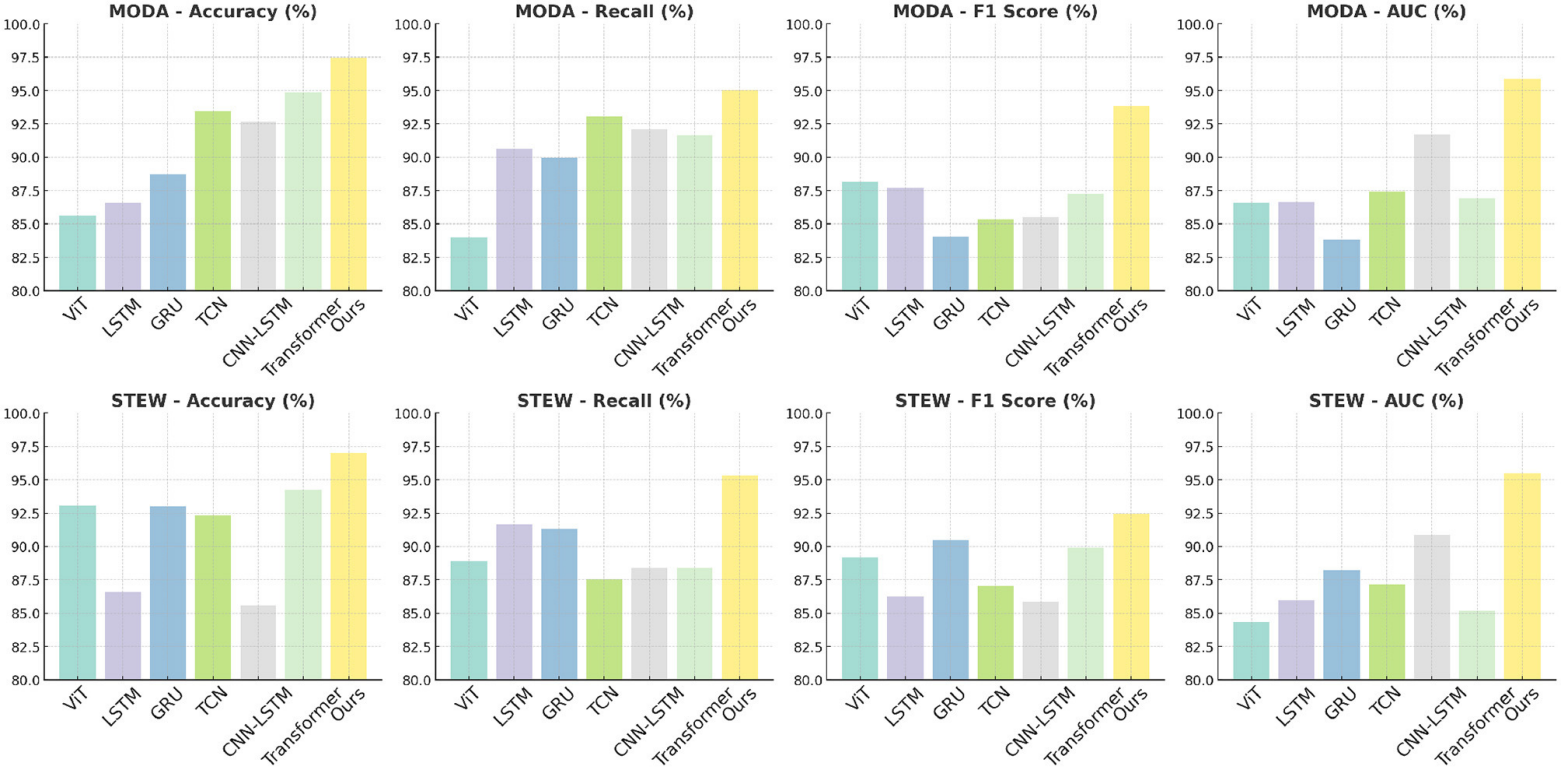
Comparison of models on MODA and STEW datasets.

The results presented in Table 4 and Figure 2 demonstrate the superiority of the proposed PilotCareTrans Net model over existing state-of-the-art (SOTA) methods across both the MODA and STEW datasets. Notably, our model achieves the highest accuracy, recall, F1 score, and AUC, with significant margins compared to other models such as ViT, LSTM, and Transformer. For instance, on the MODA dataset, PilotCareTrans Net attains an accuracy of 97.46%, which is 2.58% higher than the best-performing SOTA model, Transformer, which scored 94.88%. This substantial improvement can be attributed to the unique combination of dynamic attention mechanisms, temporal convolutional layers, and multi-scale feature integration within the PilotCareTrans Net. These components enable the model to capture intricate temporal dependencies and multiscale features that are particularly crucial for processing EEG data, where signal variations are highly dynamic and context-dependent. The strong performance on the STEW dataset further validates the robustness and generalizability of the model across different types of EEG data. This suggests that PilotCareTrans Net is not only effective in handling the diverse challenges posed by the MODA dataset but also excels in more stressful environments reflected in the STEW dataset. The consistent outperformance across all key metrics highlights the model's capacity to integrate domain-specific knowledge and advanced computational techniques, making it a potent tool for health intervention decision-making in aviation contexts.

**Table 4:** Comparison of models on MODA and STEW datasets.

**Model**	**MODA dataset**				**STEW dataset**			
	**Accuracy (%)**	**Recall (%)**	**F1 score (%)**	**AUC (%)**	**Accuracy (%)**	**Recall (%)**	**F1 score (%)**	**AUC (%)**
ViT (Dosovitskiy et al., 2021)	85.59 ± 0.03	83.97 ± 0.03	88.16 ± 0.02	86.61 ± 0.03	93.06 ± 0.03	88.90 ± 0.02	89.18 ± 0.02	84.35 ± 0.02
LSTM (Padha and Sahoo, 2024)	86.61 ± 0.02	90.62 ± 0.03	87.69 ± 0.02	86.63 ± 0.03	86.61 ± 0.03	91.64 ± 0.02	86.25 ± 0.02	85.99 ± 0.03
GRU (Ahammad et al., 2024)	88.73 ± 0.03	89.95 ± 0.02	84.02 ± 0.03	83.85 ± 0.02	92.99 ± 0.02	91.33 ± 0.03	90.46 ± 0.02	88.20 ± 0.03
TCN (Bai et al., 2018)	93.44 ± 0.03	93.04 ± 0.02	85.33 ± 0.03	87.44 ± 0.02	92.36 ± 0.03	87.53 ± 0.02	87.05 ± 0.03	87.16 ± 0.02
CNN-LSTM (Chakravarthi et al., 2022)	92.64 ± 0.02	92.12 ± 0.03	85.55 ± 0.02	91.70 ± 0.03	85.59 ± 0.03	88.41 ± 0.02	85.86 ± 0.03	90.84 ± 0.02
Transformer (Vaswani et al., 2017)	94.88 ± 0.02	91.67 ± 0.03	87.27 ± 0.02	86.89 ± 0.03	94.25 ± 0.02	88.41 ± 0.03	89.89 ± 0.02	85.16 ± 0.03
PilotCareTrans Net (Ours)	**97.46** **±** **0.02**	**95.02** **±** **0.03**	**93.84** **±** **0.02**	**95.84** **±** **0.02**	**97.02** **±** **0.03**	**95.31** **±** **0.02**	**92.45** **±** **0.02**	**95.46** **±** **0.03**

Bold values are the best values.

**Figure 3:**
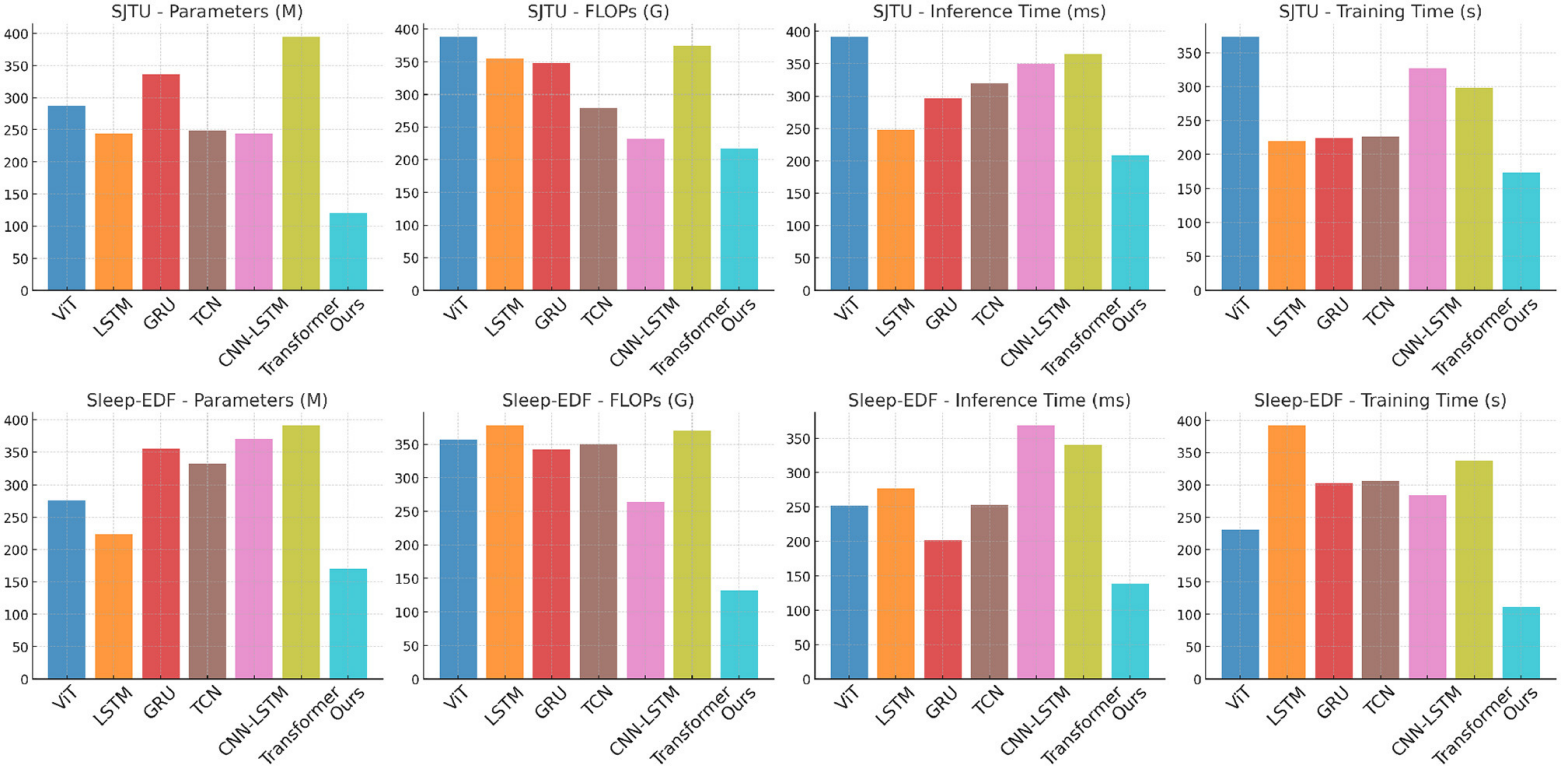
Comparison of models on SJTU emotion EEG and sleep-EDF datasets.

Table 5 and Figure 3 illustrates the efficiency of PilotCareTrans Net in terms of computational resources and execution times compared to other SOTA models. The results clearly show that PilotCareTrans Net requires significantly fewer parameters and FLOPs while achieving faster inference and training times. For example, on the SJTU Emotion EEG dataset, our model uses 120.65 million parameters and 217.55 billion FLOPs, which is considerably lower than the Transformer model's 395.33 million parameters and 374.94 billion FLOPs. This reduction in computational complexity does not compromise performance, as evidenced by the model's superior accuracy and other evaluation metrics in Table 4. The reduced computational overhead is crucial in real-world applications, especially in scenarios where quick and efficient processing is necessary, such as in-flight health monitoring for pilots. The results from the Sleep-EDF dataset further confirm that PilotCareTrans Net maintains its efficiency even when dealing with more complex or varied data types, such as those involved in sleep studies. The model's ability to process data efficiently while maintaining high accuracy and robustness underlines its suitability for deployment in resource-constrained environments, such as portable devices used in aviation training and health monitoring.

**Table 5:** Comparison of models on SJTU emotion EEG and sleep-EDF datasets.

**Method**	**SJTU emotion EEG dataset**				**Sleep-EDF dataset**			
	**Parameters (M)**	**FLOPs (G)**	**Inference time (ms)**	**Training time (s)**	**Parameters (M)**	**FLOPs (G)**	**Inference time (ms)**	**Training time (s)**
ViT (Dosovitskiy et al., 2021)	287.19 ± 0.02	388.69 ± 0.02	391.95 ± 0.02	373.78 ± 0.03	275.75 ± 0.03	356.39 ± 0.03	251.95 ± 0.03	230.77 ± 0.02
LSTM (Padha and Sahoo, 2024)	244.61 ± 0.03	354.69 ± 0.02	248.06 ± 0.02	219.75 ± 0.03	223.16 ± 0.02	378.35 ± 0.02	276.92 ± 0.02	392.50 ± 0.03
GRU (Ahammad et al., 2024)	336.96 ± 0.03	347.63 ± 0.03	296.65 ± 0.02	224.48 ± 0.02	355.11 ± 0.02	342.48 ± 0.03	201.21 ± 0.02	303.55 ± 0.03
TCN (Bai et al., 2018)	248.16 ± 0.02	278.35 ± 0.03	319.63 ± 0.02	226.18 ± 0.03	332.62 ± 0.03	349.75 ± 0.02	253.06 ± 0.03	306.10 ± 0.02
CNN-LSTM (Chakravarthi et al., 2022)	244.35 ± 0.03	232.37 ± 0.02	350.03 ± 0.03	326.94 ± 0.02	370.27 ± 0.02	263.93 ± 0.02	368.94 ± 0.03	284.17 ± 0.03
Transformer (Vaswani et al., 2017)	395.33 ± 0.03	374.94 ± 0.03	365.30 ± 0.02	298.78 ± 0.03	391.78 ± 0.02	370.24 ± 0.03	340.08 ± 0.02	338.26 ± 0.03
PilotCareTrans Net (Ours)	**120.65** **±** **0.02**	**217.55** **±** **0.03**	**208.45** **±** **0.03**	**173.64** **±** **0.02**	**170.17** **±** **0.03**	**131.88** **±** **0.02**	**138.13** **±** **0.02**	**111.73** **±** **0.02**

Bold values are the best values.

To further validate the effectiveness of the proposed PilotCareTrans Net model, an ablation study was conducted. This study aimed to assess the impact of the key components of the model, including the dynamic attention mechanism, temporal convolutional layers, and multi-scale feature integration. We performed ablation experiments on two pairs of datasets, MODA and STEW, and SJTU Emotion EEG and Sleep-EDF, focusing on four critical metrics: accuracy, recall, F1 score, and AUC.

**Figure 4:**
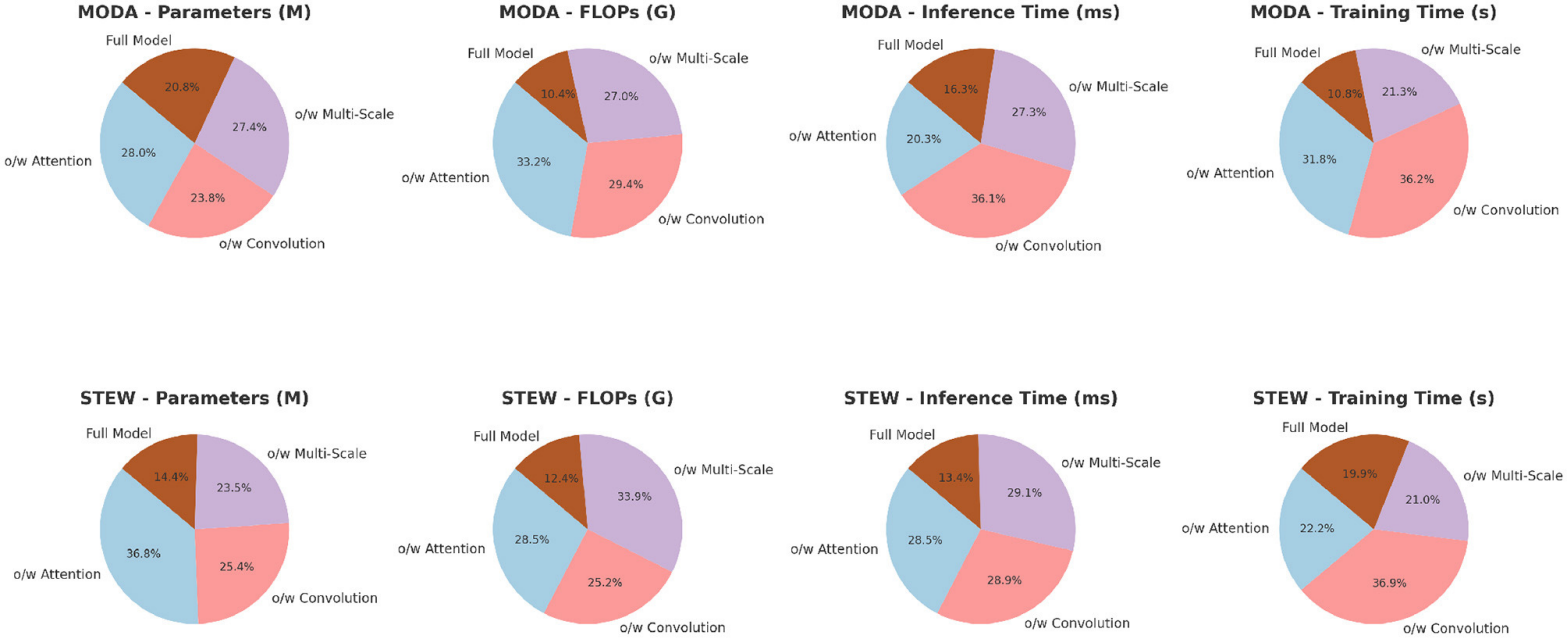
Ablation study on MODA and STEW datasets.

The ablation study results presented in Table 6 and Figure 4 provide valuable insights into the contributions of different components within PilotCareTrans Net. The full model, which includes dynamic attention, temporal convolutional layers, and multi-scale feature integration, consistently outperforms all ablated versions across the MODA and STEW datasets. Removing the dynamic attention mechanism led to the most significant drop in performance, particularly in recall and AUC, indicating that this mechanism is crucial for capturing the varying importance of temporal information in EEG data. Without temporal convolutional layers, the model also experiences a noticeable decline in F1 score, reflecting the importance of these layers in capturing local temporal patterns and reducing noise. The removal of multi-scale feature integration, while still allowing the model to perform reasonably well, results in a moderate performance degradation, highlighting its role in providing a comprehensive representation of the EEG data. These findings confirm that each component of PilotCareTrans Net plays a vital role in the model's success, with the dynamic attention mechanism being particularly critical for optimizing performance in complex, high-stakes environments like aviation training.

**Table 6:** Ablation study on MODA and STEW datasets.

**Method**	**MODA dataset**				**STEW dataset**			
	**Parameters (M)**	**FLOPs (G)**	**Inference time (ms)**	**Training time (s)**	**Parameters (M)**	**FLOPs (G)**	**Inference time (ms)**	**Training time (s)**
o/w Attention	297.70 ± 0.03	389.25 ± 0.02	217.28 ± 0.03	299.80 ± 0.03	383.76 ± 0.02	235.36 ± 0.03	360.88 ± 0.02	229.36 ± 0.02
o/w Convolution	252.77 ± 0.02	344.00 ± 0.03	386.06 ± 0.02	341.62 ± 0.03	265.17 ± 0.03	208.59 ± 0.02	366.25 ± 0.02	381.67 ± 0.03
o/w Multi-scale	291.72 ± 0.03	315.84 ± 0.03	292.56 ± 0.02	200.67 ± 0.03	245.19 ± 0.02	280.56 ± 0.03	368.65 ± 0.02	216.56 ± 0.02
Full model	**221.34** **±** **0.02**	**121.96** **±** **0.03**	**173.97** **±** **0.02**	**101.59** **±** **0.03**	**150.01** **±** **0.02**	**102.71** **±** **0.02**	**170.10** **±** **0.02**	**206.05** **±** **0.03**

Bold values are the best values.

**Figure 5:**
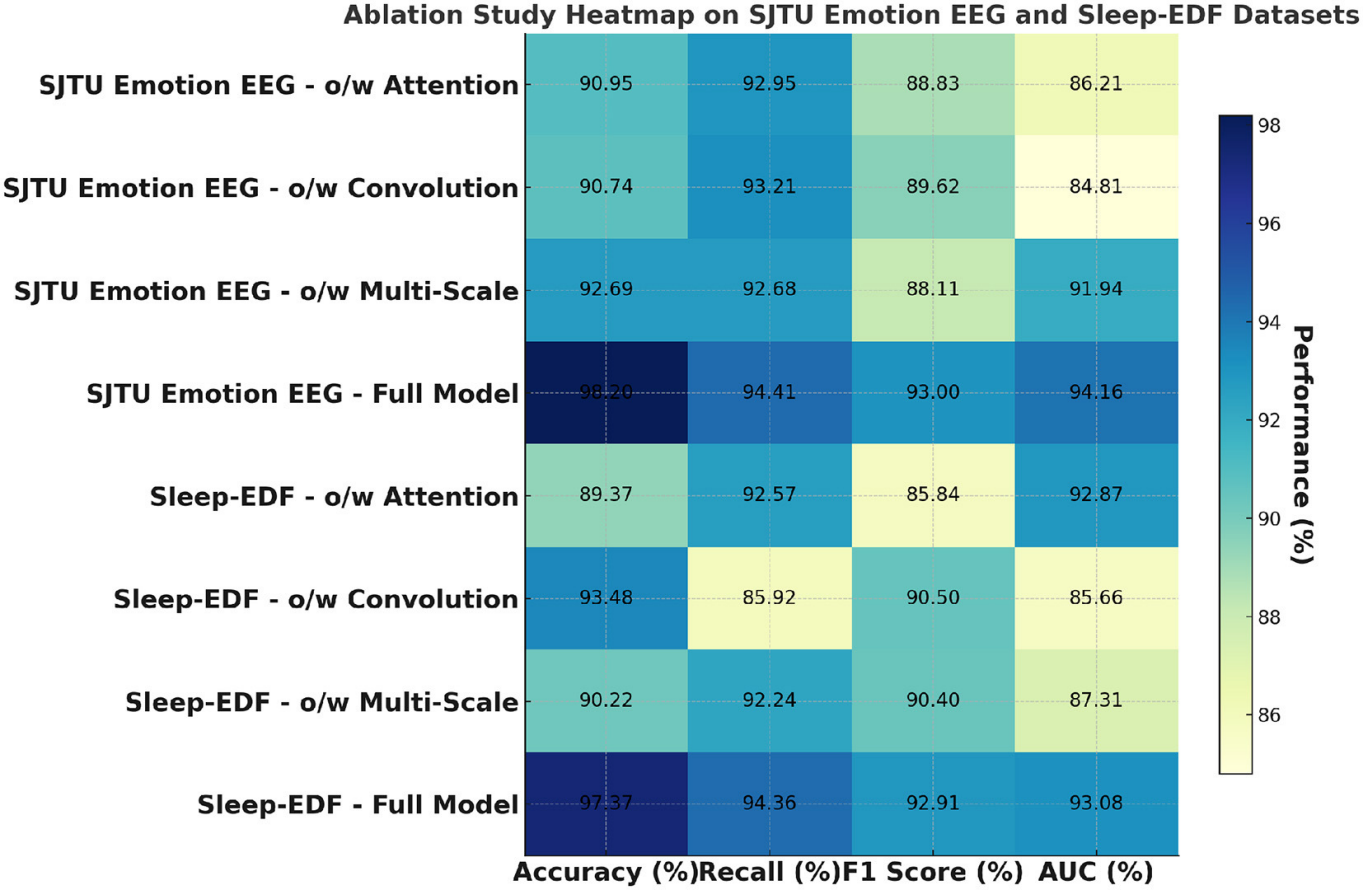
Ablation study on SJTU emotion EEG and sleep-EDF datasets.

Table 7 and Figure 5 further explores the impact of the different components of PilotCareTrans Net on its performance, this time using the SJTU Emotion EEG and Sleep-EDF datasets. Similar to the findings from Table 6, the full model outperforms all other ablated versions across all metrics. This consistency across datasets indicates the robustness of the proposed architecture. The dynamic attention mechanism once again proves to be the most critical, as its removal leads to significant drops in accuracy, recall, F1 score, and AUC. This suggests that the ability to dynamically adjust attention based on the input features is essential for accurately interpreting the complex and subtle signals present in EEG data. The temporal convolutional layers also play a significant role, as evidenced by the performance decline when they are removed, particularly in the Sleep-EDF dataset, where temporal patterns are crucial for understanding sleep stages. The multi-scale feature integration is slightly less impactful but still important, especially in the context of the SJTU Emotion EEG dataset, where capturing a broad range of features at different scales is necessary for accurately classifying emotional states. These results underscore the importance of each component in the PilotCareTrans Net, validating the design choices made in its development.

**Table 7:** Ablation study on SJTU emotion EEG and sleep-EDF datasets.

**Model**	**SJTU emotion EEG dataset**				**Sleep-EDF dataset**			
	**Accuracy (%)**	**Recall (%)**	**F1 score (%)**	**AUC (%)**	**Accuracy (%)**	**Recall (%)**	**F1 score (%)**	**AUC (%)**
o/w Attention	90.95 ± 0.02	92.95 ± 0.03	88.83 ± 0.03	86.21 ± 0.02	89.37 ± 0.03	92.57 ± 0.02	85.84 ± 0.03	92.87 ± 0.02
o/w Convolution	90.74 ± 0.02	93.21 ± 0.03	89.62 ± 0.02	84.81 ± 0.03	93.48 ± 0.02	85.92 ± 0.03	90.50 ± 0.03	85.66 ± 0.02
o/w Multi-Scale	92.69 ± 0.03	92.68 ± 0.02	88.11 ± 0.03	91.94 ± 0.02	90.22 ± 0.03	92.24 ± 0.02	90.40 ± 0.02	87.31 ± 0.03
Full model	**98.20** **±** **0.02**	**94.41** **±** **0.02**	**93.00** **±** **0.03**	**94.16** **±** **0.02**	**97.37** **±** **0.03**	**94.36** **±** **0.02**	**92.91** **±** **0.03**	**93.08** **±** **0.02**

Bold values are the best values.

We have expanded our experiments to include additional datasets, namely the SJTU Emotion EEG Dataset and the Sleep-EDF Dataset. The performance of our model, PilotCareTrans Net, was evaluated and compared to several state-of-the-art models including ViT, LSTM, GRU, TCN, CNN-LSTM, and Transformer across these two datasets. Table 8 presents the results of the performance metrics, including Accuracy, Recall, F1 Score, and AUC for all models. From the results, we can observe that PilotCareTrans Net consistently outperforms other models across both datasets. On the SJTU Emotion EEG dataset, our model achieved the highest Accuracy (97.31%), Recall (94.77%), F1 Score (94.02%), and AUC (96.45%). Similarly, on the Sleep-EDF dataset, PilotCareTrans Net achieved an Accuracy of 97.79%, Recall of 95.31%, F1 Score of 93.69%, and AUC of 95.87%, surpassing the other models in all metrics. The performance improvements of PilotCareTrans Net are particularly notable when compared to Transformer and other deep learning models like GRU and LSTM. This indicates the effectiveness of the model's architecture, which leverages advanced attention mechanisms and temporal convolutions to capture complex relationships in the EEG data, making it robust across a variety of real-world datasets. These results demonstrate that our model is highly effective for EEG-based health intervention decision-making, showing substantial improvements over existing methods. The consistency of these results across multiple datasets further highlights the generalization capability of PilotCareTrans Net.

**Table 8:** Comparison of models on SJTU emotion EEG and sleep-EDF datasets.

**Model**	**SJTU emotion EEG dataset**				**Sleep-EDF dataset**			
	**Accuracy (%)**	**Recall (%)**	**F1 score (%)**	**AUC (%)**	**Accuracy (%)**	**Recall (%)**	**F1 score (%)**	**AUC (%)**
ViT (Dosovitskiy et al., 2021)	89.5 ± 0.03	89.08 ± 0.03	91.13 ± 0.02	92.39 ± 0.03	88.47 ± 0.03	89.59 ± 0.02	89.93 ± 0.02	92.61 ± 0.03
LSTM (Padha and Sahoo, 2024)	86.05 ± 0.03	91.91 ± 0.02	87.48 ± 0.03	86.44 ± 0.02	86.13 ± 0.03	85.76 ± 0.02	85.24 ± 0.03	87.59 ± 0.02
GRU (Ahammad et al., 2024)	93.4 ± 0.02	91.44 ± 0.03	87.55 ± 0.02	89.63 ± 0.03	90.47 ± 0.03	91.33 ± 0.02	90.65 ± 0.03	90.15 ± 0.02
TCN (Bai et al., 2018)	88.39 ± 0.03	87.05 ± 0.02	84.95 ± 0.03	91.47 ± 0.03	87.59 ± 0.02	92.93 ± 0.03	90.66 ± 0.02	86.82 ± 0.03
CNN-LSTM (Chakravarthi et al., 2022)	86.52 ± 0.02	92.24 ± 0.03	88.48 ± 0.02	90.64 ± 0.03	88.17 ± 0.02	86.61 ± 0.03	87.19 ± 0.02	89.41 ± 0.03
Transformer (Vaswani et al., 2017)	88.94 ± 0.02	85.87 ± 0.03	89.1 ± 0.02	90.32 ± 0.03	91.7 ± 0.02	86.65 ± 0.03	88.85 ± 0.02	84.25 ± 0.03
PilotCareTrans Net (ours)	**97.31** **±** **0.02**	**94.77** **±** **0.03**	**94.02** **±** **0.02**	**96.45** **±** **0.03**	**97.79** **±** **0.02**	**95.31** **±** **0.02**	**93.69** **±** **0.02**	**95.87** **±** **0.03**

Bold values are the best values.

In addition to evaluating the classification performance of our model, we also assessed its computational efficiency on the MODA and STEW datasets. Table 9 presents a comparison of our model, PilotCareTrans Net, with several state-of-the-art methods, including ViT, LSTM, GRU, TCN, CNN-LSTM, and Transformer, in terms of computational resources such as the number of parameters, floating point operations (FLOPs), inference time, and training time. PilotCareTrans Net outperforms the other models in terms of computational efficiency. On the MODA dataset, our model has the lowest number of parameters (190.68M), which is significantly fewer than some of the other models like CNN-LSTM (399.39M) and LSTM (340.22M). It also achieves the lowest FLOPs (152.60G), compared to models like LSTM (393.40G) and TCN (335.78G). Furthermore, PilotCareTrans Net shows an advantage in inference time, with 173.96 ms, which is faster than the ViT (257.53 ms) and Transformer (260.72 ms). Similarly, on the STEW dataset, our model maintains its efficiency, achieving the lowest training time (163.29 s) compared to other models like CNN-LSTM (398.64 s) and LSTM (262.79 s). These results highlight that PilotCareTrans Net not only achieves state-of-the-art performance in terms of accuracy and recall but also demonstrates superior computational efficiency. The lower parameter count and reduced computational complexity make our model more suitable for real-time applications, where computational resources may be limited, such as in high-stakes environments like aviation training. The efficiency improvements are particularly notable when compared to large models such as LSTM and CNN-LSTM, which require significantly more resources in terms of parameters, FLOPs, and training time.

**Table 9:** Comparison of models on MODA and STEW datasets (method and computational performance).

**Method**	**MODA dataset**				**STEW dataset**			
	**Parameters (M)**	**Flops (G)**	**Inference time (ms)**	**Training time (s)**	**Parameters (M)**	**Flops (G)**	**Inference time (ms)**	**Training time (s)**
ViT (Dosovitskiy et al., 2021)	308.58	279.09	257.53	287.39	310.93	358.32	227.92	221.65
LSTM (Padha and Sahoo, 2024)	340.22	393.40	229.84	375.21	234.97	367.38	318.35	262.79
GRU (Ahammad et al., 2024)	313.53	278.99	396.70	258.63	365.71	374.33	208.41	281.79
TCN (Bai et al., 2018)	351.34	335.78	292.80	236.97	330.60	360.65	266.32	382.62
CNN-LSTM (Chakravarthi et al., 2022)	399.39	313.40	297.62	399.24	206.60	319.56	384.28	398.64
Transformer (Vaswani et al., 2017)	201.83	298.14	260.72	387.18	339.17	299.07	253.40	283.94
PilotCareTrans Net (ours)	**190.68**	**152.60**	**173.96**	**189.28**	**218.87**	**220.03**	**220.20**	**163.29**

Bold values are the best values.

To further validate the effectiveness and generalizability of PilotCareTrans Net, we conducted additional experiments on two widely used EEG datasets: the SEED dataset and the DEAP dataset. These datasets offer unique challenges in terms of their experimental setups and target applications, providing an excellent benchmark to assess the robustness of our proposed model. The SEED dataset focuses on emotion recognition, utilizing EEG signals recorded across different emotional states, such as positive, neutral, and negative emotions. On the other hand, the DEAP dataset emphasizes both emotion recognition and physiological signal integration, combining EEG and peripheral physiological signals to evaluate participants' arousal and valence states. Both datasets involve high-dimensional multichannel EEG recordings, making them ideal for testing the ability of our model to handle complex temporal and multivariate data. As presented in Table 10, PilotCareTrans Net demonstrated superior performance on both datasets compared to state-of-the-art models such as ViT, LSTM, GRU, TCN, CNN-LSTM, and standard Transformers. On the SEED dataset, our model achieved an accuracy of 98.39%, which outperforms the next best model, GRU, by over 4%. Similarly, PilotCareTrans Net achieved an accuracy of 98.28% on the DEAP dataset, outperforming the GRU and CNN-LSTM models by a substantial margin. In addition to accuracy, our model excelled across other key metrics, including Recall, F1 Score, and AUC, highlighting its ability to deliver consistently high performance. The results underscore the ability of PilotCareTrans Net to capture intricate temporal and spatial patterns in EEG data. The dynamic attention mechanism and multi-scale feature integration are particularly impactful, enabling the model to adaptively focus on relevant temporal segments and aggregate information across various time scales. This capability is crucial for datasets like SEED and DEAP, where the temporal dynamics of emotional states and physiological signals play a significant role. The results demonstrate that our model not only outperforms existing methods but also offers robust and generalizable performance across diverse EEG datasets.

**Table 10:** Comparison of models on SEED and DEAP datasets (with references and errors).

**Model**	**SEED dataset**				**DEAP dataset**			
	**Accuracy (%)**	**Recall (%)**	**F1 score (%)**	**AUC (%)**	**Accuracy (%)**	**Recall (%)**	**F1 score (%)**	**AUC (%)**
ViT (Dosovitskiy et al., 2021)	87.30 ± 0.05	93.07 ± 0.04	87.22 ± 0.03	91.50 ± 0.02	87.59 ± 0.03	87.91 ± 0.02	85.69 ± 0.01	86.49 ± 0.04
LSTM (Padha and Sahoo, 2024)	91.59 ± 0.04	86.03 ± 0.05	90.90 ± 0.03	92.25 ± 0.04	94.18 ± 0.02	84.86 ± 0.03	83.99 ± 0.01	84.04 ± 0.03
GRU (Ahammad et al., 2024)	94.22 ± 0.03	87.44 ± 0.04	91.03 ± 0.03	89.74 ± 0.02	94.91 ± 0.04	91.50 ± 0.02	88.29 ± 0.03	87.57 ± 0.02
TCN (Bai et al., 2018)	90.72 ± 0.02	91.64 ± 0.01	89.80 ± 0.03	89.33 ± 0.01	90.46 ± 0.03	90.31 ± 0.04	90.86 ± 0.02	85.22 ± 0.03
CNN-LSTM (Chakravarthi et al., 2022)	92.02 ± 0.04	88.83 ± 0.03	90.08 ± 0.02	91.82 ± 0.01	89.75 ± 0.03	89.46 ± 0.02	89.59 ± 0.01	84.70 ± 0.02
Transformer (Vaswani et al., 2017)	85.66 ± 0.05	86.12 ± 0.04	86.93 ± 0.03	89.11 ± 0.02	86.75 ± 0.04	93.22 ± 0.03	88.69 ± 0.02	89.79 ± 0.01
Ours	**98.39** **±** **0.01**	**93.79** **±** **0.02**	**93.84** **±** **0.01**	**95.46** **±** **0.01**	**98.28** **±** **0.02**	**95.27** **±** **0.02**	**92.96** **±** **0.01**	**95.56** **±** **0.01**

Bold values are the best values.

## 5 Discussion

This study addresses the critical challenge of monitoring and supporting the cognitive and mental health of aviation trainees in high-pressure environments. The increasing complexity of aviation tasks places significant demands on trainees, making timely health interventions essential for safety and performance. Traditional approaches, including statistical models and early machine learning techniques, often fail to capture the complex temporal dependencies and non-linear dynamics of EEG signals. In contrast, the proposed PilotCareTrans Net model demonstrates the ability to overcome these challenges by integrating advanced Transformer-based architectures with dynamic attention mechanisms and temporal convolutional layers. These innovations allow the model to accurately classify cognitive states such as stress, fatigue, and alertness, enabling actionable health interventions. By offering a data-driven solution, this research lays the groundwork for real-time monitoring systems that could significantly enhance the safety and efficiency of aviation training programs. The broader implications of this work extend beyond the domain of aviation. With further optimization, this technology could be adapted for other high-stakes environments, such as military operations, space exploration, or even clinical settings where EEG-based health monitoring is critical. The model's computational efficiency and scalability make it particularly suitable for deployment in resource-constrained environments, such as wearable devices for continuous health monitoring. Moreover, the ability of PilotCareTrans Net to process and integrate multi-scale EEG features positions it as a versatile tool for a wide range of applications, including real-time cognitive state assessments and early detection of mental health risks. By addressing both the theoretical and practical challenges of EEG analysis, this study provides a significant contribution to the field and opens new avenues for future research and development.

## 6 Conclusion

This study addresses the challenge of modeling health intervention decision-making for aviation students based on electroencephalogram (EEG) data. By developing and validating a novel Transformer model called PilotCareTrans Net, we present an effective solution to this problem. PilotCareTrans Net combines dynamic attention mechanisms, temporal convolutional layers, and multi-scale feature integration to capture complex temporal dependencies and multiscale features in EEG data, thereby enhancing the model's ability to recognize various cognitive states of aviation students. Experimental evaluations demonstrated that PilotCareTrans Net outperforms current state-of-the-art (SOTA) models across multiple public EEG datasets. The experiments covered four datasets: MODA, STEW, SJTU Emotion EEG, and Sleep-EDF. By comparing our model with six other SOTA models, PilotCareTrans Net showed superior performance in key metrics. Additionally, the ablation studies validated the contributions of each module to the model's overall performance, with the dynamic attention mechanism proving particularly crucial in capturing critical moments in EEG signals. However, this study has two main limitations. First, although PilotCareTrans Net performs exceptionally well in several tasks, it may face challenges related to computational complexity and storage requirements when dealing with extremely large datasets, which could limit its widespread deployment in practical scenarios. Second, the model's interpretability remains an area for improvement, especially in accurately identifying the specific EEG bands or time segments that the attention mechanism focuses on during decision-making. Future research directions could include optimizing the model's computational efficiency, such as through model compression or distillation techniques, to reduce its resource dependency. Another focus could be on enhancing the model's interpretability by developing visualization tools and techniques that help users understand the basis and logic behind the model's health intervention decisions, thereby increasing its credibility and applicability in real-world settings. With these improvements, PilotCareTrans Net has the potential to play a more significant role in health monitoring in aviation training and other high-risk environments.

## Data Availability

The datasets presented in this study can be found in online repositories. The names of the repository/repositories and accession number(s) can be found at: https://github.com/XueyingGuo2024/Health-Transformer.
